# Comparison of Outcomes and Process of Care for Patients Treated at Hospitals Dedicated for COVID-19 Care vs Other Hospitals

**DOI:** 10.1001/jamanetworkopen.2022.0873

**Published:** 2022-03-03

**Authors:** Zachary R. Bergman, Michael Usher, Andrew Olson, Jeffrey G. Chipman, Melissa E. Brunsvold, Greg Beilman, Christopher Tignanelli, Elizabeth R. Lusczek

**Affiliations:** 1 Department of Surgery, University of Minnesota, Minneapolis; 2 Department of Medicine, University of Minnesota, Minneapolis; 3 Department of Pediatrics, University of Minnesota, Minneapolis; 4 M. Health Fairview Health System Management, Minneapolis, Minnesota

## Abstract

**Importance:**

Early in the SARS-CoV-2 pandemic, the M Health Fairview Hospital System established dedicated hospitals for establishing cohorts and caring for patients with COVID-19, yet the association between treatment at COVID-19–dedicated hospitals and mortality and complications is not known.

**Objective:**

To analyze the mortality rate and complications associated with treatment at the COVID-19–dedicated hospitals.

**Design, Setting, and Participants:**

This retrospective cohort study evaluated data prospectively collected from March 1, 2020, through June 30, 2021, from 11 hospitals in Minnesota, including 2 hospitals created solely to care for patients with COVID-19. Data obtained included demographic characteristics, treatments, and outcomes of interest for all patients with a confirmed COVID-19 infection admitted to this hospital system during the study period.

**Exposures:**

Patients were grouped based on whether they received treatment from 1 of the 2 COVID-19–dedicated hospitals compared with the remainder of the hospitals within the hospital system.

**Main Outcomes and Measures:**

Multivariate analyses, including risk-adjusted logistic regression and propensity score matching, were performed to evaluate the primary outcome of in-hospital mortality and secondary outcomes, including complications and use of COVID-specific therapeutics.

**Results:**

There were 5504 patients with COVID-19 admitted during the study period (median age, 62.5 [IQR, 45.0-75.6] years; 2854 women [51.9%]). Of these, 2077 patients (37.7%) (median age, 63.4 [IQR, 50.7-76.1] years; 1080 men [52.0%]) were treated at 1 of the 2 COVID-19–dedicated hospitals compared with 3427 (62.3%; median age, 62.0 [40.0-75.1] years; 1857 women (54.2%) treated at other hospitals. The mortality rate was 11.6% (n = 241) at the dedicated hospitals compared with 8.0% (n = 274) at the other hospitals (*P* < .001). However, risk-adjusted in-hospital mortality was significantly lower for patients in the COVID-19–dedicated hospitals in both the unmatched group (n = 2077; odds ratio [OR], 0.75; 95% CI, 0.59-0.95) and the propensity score–matched group (n = 1317; OR, 0.78; 95% CI, 0.58-0.99). The rate of overall complications in the propensity score–matched group was significantly lower (OR, 0.81; 95% CI, 0.66-0.99) and the use of COVID-19–specific therapeutics including deep vein thrombosis prophylaxis (83.9% vs 56.9%; *P* < .001), high-dose corticosteroids (56.1% vs 22.2%; *P* < .001), remdesivir (61.5% vs 44.5%; *P* < .001), and tocilizumab (7.9% vs 2.0; *P* < .001) was significantly higher.

**Conclusions and Relevance:**

In this cohort study, COVID-19–dedicated hospitals had multiple benefits, including providing high-volume repetitive treatment and isolating patients with the infection. This experience suggests improved in-hospital mortality for patients treated at dedicated hospitals owing to improved processes of care and supports the use of establishing cohorts for future pandemics.

## Key Points

**Question:**

Is treatment of COVID-19 at a dedicated hospital associated with improved care processes and outcomes?

**Findings:**

In this cohort study of 5504 patients with COVID-19, lower mortality rates were found in dedicated COVID-19 hospitals vs other hospitals.

**Meaning:**

Results of this study suggest that treatment at dedicated COVID-19 hospitals may be associated with reducing in-hospital mortality; this model may be useful during future pandemics.

## Teaser

This cohort study compares the mortality rate and complications associated with treatment in hospitals dedicated to the care of patients with COVID-19 vs other hospitals.

## Introduction

Hospitals or communities dedicated to isolating infectious disease have been used throughout history to decrease contagious spread. Whole cities established to isolate those infected with leprosy were prominent for centuries.^1,2^ In the 1800s, tuberculosis sanatoriums isolated patients to prevent additional spread and were often built in temperate locations that provided a therapeutic benefit.^3^ More recently, during the Middle Eastern respiratory syndrome outbreak, South Korea established hospitals dedicated to patients with this infection to decrease the rate of in-hospital transmission of the highly contagious virus.^4^

As news of the emerging SARS-CoV-2 pandemic spread in early 2020, M Health Fairview, a large academic health care system with 11 hospitals in Minnesota, rapidly converted a previous long-term acute care hospital into a dedicated COVID-19 hospital. Bethesda Hospital in Saint Paul was a 90-bed community hospital founded in 1883 and transformed into a long-term acute care hospital in 1989. In March 2020, it was converted to a hospital focused on patients with COVID-19, with 35 intensive care unit (ICU) beds and 55 general care beds.^5,6^ Extensive building modifications were made, including installing telemetry capabilities, creating negative airflow in patient rooms with HEPA-filter fans, and updating interventional radiologic and procedural suites. The hospital was staffed by a combination of medical and surgical intensivists, hospitalists, and ICU and general floor nurses.^7^ In November 2020, due to increasing demand, care transitioned from Bethesda to Saint Joseph's Hospital, also in Saint Paul, with a capacity of 41 ICU beds and 68 general floor beds.^8^ In combination, these hospitals served to provide care for the patients most severely ill with COVID-19 and increase surge capacity for the M Health Fairview health care system through June 2021.

The creation of a COVID-19–specific hospital was proposed to provide benefit to both health care professionals and patients. Protocols were put in place to ensure health care professionals were able to use proper personal protection with all patients with easy availability, even in times of generalized shortage. We believed that this strategy, in combination with the isolation of patients with COVID-19 from those without COVID-19, would decrease rates of in-hospital transmission. In addition, by focusing specifically on COVID-19, clinicians were able to closely follow evolving treatment recommendations. Protocols to ensure delivery of beneficial therapeutics to the patients were implemented rapidly within the COVID-19–dedicated hospital model to provide the most up-to-date care, in part by use of dedicated ordersets and documentation templates. The changes were based on landmark studies, such as the RECOVERY Trial, which demonstrated improved survival with the use of high-dose corticosteroids,^9^ and the Beigel et al^10^ trial, which demonstrated improved recovery time for patients receiving remdesivir.

There was a focus on research from the outset of the pandemic as both COVID-19–dedicated hospitals served as sites for major trials. In addition, systemwide outcomes from all patients who tested positive for SARS-CoV-2 within the M Health Fairview health system were added to a large prospective database. There have been a few brief reports published describing the process that similar health care systems worldwide have used to establish COVID-19–dedicated hospitals.^4,11,12^ There has also been a report on the use of interhospital transfer to the COVID-19–dedicated hospitals, the association between use of these hospitals and quality care measures, and health care professional satisfaction within the M Health Fairview system.^13^ However, to our knowledge, there has not been a large-scale evaluation of the outcomes of this practice. Herein we present a comparison of survival metrics and outcomes comparing patients receiving care at COVID-19–dedicated hospitals with those cared for at the other facilities within the M Health Fairview hospital system.

## Methods

### Study Design and Patient Population

This cohort study was an evaluation of prospectively collected data from patients within the M Health Fairview Hospital System of 11 hospitals from March 1, 2020, through June 30, 2021. Consecutive patients aged 18 years or older with SARS-CoV-2 infection confirmed by reverse transcription polymerase chain reaction who were admitted to the hospital were included. Patients were excluded if they had indicated in their electronic health record they wished to opt-out of research. The study was approved by the internal review board at the University of Minnesota. Patient informed consent was not required owing to the deidentified patient data. The database, containing only deidentified patient information, includes home medications, laboratory values, clinic visits, social history, and patient demographic characteristics (age, sex, race and ethnicity, language spoken, zip code, and socioeconomic status indicators). The hospitalization data include all laboratory values, vital signs, orders, medications, complications, length of stay, and disposition. Race and ethnicity are self-reported and were included to confirm the propensity matching. This study followed the Strengthening the Reporting of Observational Studies in Epidemiology (STROBE) reporting guideline.

### Outcomes

The primary outcome was in-hospital mortality. The hospital quality metrics that were evaluated were the rate of deep vein thrombosis (DVT) prophylaxis and the use of COVID-19 therapeutics including the antiviral remdesivir, anti-IL-6 antibody tocilizumab, and dexamethasone (high dose [≥6 mg/d] vs low dose [<6 mg/d]). Evaluated in-hospital complications included ventilator or hospital-acquired pneumonia, bacteremia, urinary tract infections, stroke, acute myocardial infarction, pulmonary embolism, DVT, acute kidney injury, liver failure, and bleeding events. A patient was considered to have a complication if they developed 1 or more of these events. Additional outcomes included ICU admission rates, rate of mechanical ventilation, and hospital and ICU length of stay (LOS).

### Statistical Analysis

Data analysis was performed using a combination of Stata, version 16 (StataCorp LLC) and R, version 3.6.3 (R Foundation for Statistical Computing). Treatment groups were determined by whether the patient received care at either COVID-19–dedicated hospital (Bethesda Hospital or Saint Joseph’s Hospital; COVID-19–dedicated group), or another system hospital (other hospitals group). Normally distributed data are reported as mean (SD) and nonnormally distributed data as median, with IQR for continuous variables and proportions for categorical variables. Continuous variables were compared between treatment groups using a *t* test for normally distributed variables and Wilcoxon rank-sum test for nonnormally distributed variables. Categorical characteristics and outcomes were compared between treatment groups using a Pearson χ^2^ test or Fisher exact test, depending on sample size. Two sided, unpaired tests were used in determining significance at a threshold of *P* < .05.

Imputation was performed in Stata, using predictive mean matching to impute 5 data sets. To determine whether treatment at a COVID-19–dedicated hospital was independently associated with reduced mortality, multivariate models for mortality were developed with the least absolute shrinkage and selection operator (LASSO) method.^14^ The variables utilized for the LASSO model were a combination of previously identified risk factors^15,16,17,18^ as well as variables identified by a group of intensivists and hospitalists with significant experience caring for patients at the COVID-19 dedicated hospitals. Physicians were asked to identify demographic, clinical, and laboratory variables that in their clinical experience may be associated with in-hospital mortality. Variables that were significantly associated with in-hospital mortality in the LASSO model were utilized for the logistic regression as well as creating propensity score–matched groups (eTable 1 in the Supplement).

One of the 5 imputed data sets was selected at random to use to perform propensity score matching. Propensity score–matched mixed-effects logistic regression was performed, stratified by in-hospital mortality. Propensity scores were estimated with logistic regression with variables selected by the LASSO logistic model using a common caliper set at 0.05. Even distribution of propensity scores was confirmed between matched groups, with standardized differences less than 0.1 for all confounding variables. Successful matching was confirmed by comparing demographic, clinical, and laboratory data between the 2 treatment groups.

Logistic regression was used to compare in-hospital mortality and complications. In the unmatched group, the pooled imputed data sets were analyzed by adjusting for the variables that were identified by LASSO to be associated with in-hospital mortality. In the propensity score–matched group, we adjusted for d-dimer level, because this difference remained significant after matching.

Sensitivity analyses were performed with logistic regression on the pooled group of imputed data sets. Three sensitivity analyses were conducted on the full unmatched cohort by excluding key subgroups of patients. The excluded groups were patients with hospital LOS less than or equal to 3 days, patients treated within the first 3 months of the COVID-19–dedicated hospital’s operation, and patients who died within 24 hours of admission. A fourth sensitivity analysis added exact matching by admission month to the propensity score matching to account for changes in treatment approaches over time. A fifth sensitivity analysis performed the logistic regression on the unmatched cohort while excluding laboratory value variables because these variables accounted for nearly all of the imputed data (95.4%).

## Results

### System-Wide Outcomes

There was a total of 45 609 patients who tested positive for SARS-CoV-2 in the system, and 5504 of these patients (2854 women [51.9%] and 2650 men [48.1%]; median age, 62.5 [IQR, 45.0-75.6] years) were admitted and included in the study. The overall survival to discharge for admitted patients was 90.8%; the overall in-hospital mortality rate was 9.2%. Intensive care unit admissions accounted for 26.9% (n = 1478) of all admissions with a survival to discharge in this subset of patients of 75.1% (n = 1110). A total of 539 hospitalized patients (9.8%) required intubation and mechanical ventilation. The median hospital LOS for all patients was 5.0 (IQR, 2.8-9.2) days. Patients in the ICU had a median ICU LOS of 5.4 (IQR, 2.0-12.6) days and a median hospital LOS of 10.9 (IQR, 5.7-19.1) days.

### Univariate Analysis and Unmatched Cohort Survival

**Figure 1.**
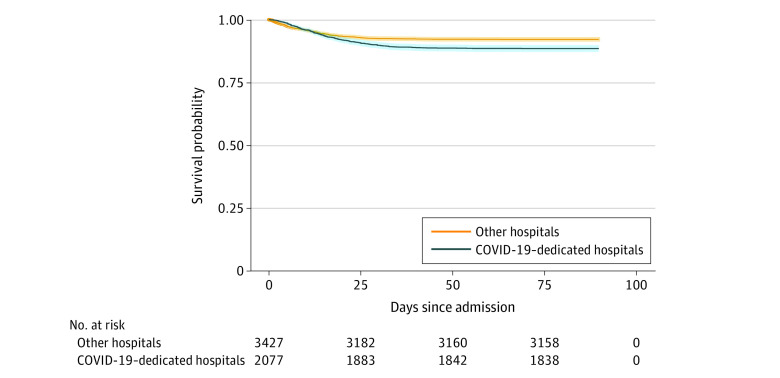
Survival for the Unmatched Group of Patients The overall survival rate was 88.4% at the COVID-19–dedicated hospitals compared with 92.0% at the other hospitals (*P* < .001). The shaded areas represent the 95% CIs.

The treatment groups included 2077 patients (37.7%; 997 women [48.0%] and 1080 men [52.0%]; median age, 63.4 [IQR, 50.7-76.1] years) in the COVID-19–dedicated hospitals and 3427 patients (62.3%; 1857 women [54.2%] and 1570 men [45.8%]; median age, 62.0 [IQR, 40.0-75.1] years) in other hospitals. Other demographic characteristics of the patient population are reported in the Table. The other hospitals vs COVID-19–dedicated hospitals were significantly different in nearly all evaluated demographic, clinical, and laboratory data, including median BMI (29.4 [IQR, 25.0-34.3] vs 30 [IQR, 25.3-35.6]; *P*<.001); non-English speaking (15.6% [n = 533] vs 18.2% [n = 378]; *P* = .01), female (54.2% [n = 1857] vs 48.0% [n = 997]; *P*<.001), median Elixhauser Comorbidity Index score (5 [IQR, 3-9] vs 6 [IQR, 3-9]; *P*<.001), use of home angiotensin-converting enzyme inhibitors (11.7% [n = 400] vs 8.5% [n = 177]; *P*<.001), use of home inhaled corticosteroids (7.9% [n = 271] vs 9.9% [n = 205]; *P*<.001), median minimum oxygen saturation as measured by pulse oximetry (91% [IQR, 87%-94%] vs 89% [IQR, 85%-92%]; *P*<.001), median maximum respiratory rate (24 [IQR, 20-32] vs 28 [IQR, 22-38] breaths/min; *P*<.001), median maximum temperature (37.4 [IQR, 37.1-38.2] vs 37.7 [37.2 − 38.5] °C; *P*<.001), highest creatinine level (0.85 [IQR, 0.69-1.13] vs 0.87 [IQR, 0.73-1.16] mg/dL; *P*<.001 [to convert to micromoles per liter, multiply by 88.4]), highest d-dimer level (1.01 [IQR, 0.6-2.08] vs 0.97 g/L [IQR, 0.58-1.76] g/L; *P* = 0.008 [to convert to nanomoles per liter, multiply by 5.476]), highest C-reactive protein level, 66 [IQR, 27.8-1.24] vs 80 [IQR, 40-138] mg/L; *P*<0.001 [to convert to milligrams per liter, multiply by 10]) lowest absolute lymphocyte count (900 [IQR, 600-1300] vs 800 [IQR, 600-1200] cells/μL; *P* = .03 [to convert to ×10^9^ per liter, multiply by 0.001]), rate of mechanical ventilation (6.1% [n = 209] vs 15.9% [n = 330]; *P *<.001), and rate of ICU admission (18.2% [n = 624] vs 41.1% [n = 854]; *P*<.001). More severely ill patients received treatment at the COVID-19 dedicated hospitals. The dedicated hospitals had a higher rate of ICU admissions (41.1% vs 18.2%; *P* < .001). Both median hospital LOS (7.2 vs 3.9 days; *P* < .001) and ICU LOS (6.9 vs 3.6 days; *P* < .001) were significantly longer in the COVID-19–dedicated hospital group. Kaplan-Meier survival curves evaluating in-hospital mortality for the unmatched group of patients are shown in Figure 1. The overall survival was 88.4% at the dedicated hospitals compared with 92.0% at the other hospitals (*P* < .001). The in-hospital mortality rate was 11.6% (n = 241) at the dedicated hospitals compared with 8.0% (n = 274) at the other hospitals (*P* < .001).

**Table.** Patient Characteristics

Variable	No. (%)			*P* value
	Overall (N = 5504)	Other hospitals (n = 3427)	COVID-19–dedicated hospitals (n = 2077)	
Age, median (IQR), y	62.5 (45.0-75.6)	62.0 (40.0-75.1)	63.4 (50.7-76.1)	<.001
BMI, median (IQR)	29.6 (25.1-34.7)	29.4 (25.0-34.3)	30 (25.3-35.6)	<.001
Non-English speaking	911 (16.6)	533 (15.6)	378 (18.2)	.01
Race^a^				
Asian	619 (11.2)	328 (9.6)	291 (14.0)	<.001
Black	660 (12.0)	416 (12.)	244 (11.7)	
Hispanic	358 (6.5)	202 (5.9)	156 (7.5)	
White	3664 (66.6)	2354 (68.7)	1310 (63.1)	
Declined to report	106 (1.9)	73 (2.1)	33 (1.6)	
Other	97 (1.8)	54 (1.6)	43 (2.1)	
Sex				
Female	2854 (51.9)	1857 (54.2)	997 (48.0)	<.001
Male	2650 (48.1)	1570 (45.8)	1080 (52.0)	
Comorbidities/home medications				
Elixhauser Comorbidity Index, median (IQR)	5 (3-9)	5 (3-9)	6 (3-9)	<.001
Preadmission medication				
ACE inhibitor	577 (10.5)	400 (11.7)	177 (8.5)	<.001
Metformin	417 (7.6)	265 (7.7)	152 (7.3)	.57
Insulin	477 (8.7)	313 (9.1)	164 (7.9)	.11
Inhaled corticosteroid	476 (8.6)	271 (7.9)	205 (9.9)	.01
Admission data within 48-72 h, median (IQR)				
Minimum Spo_2_, %	90 (86-93)	91 (87-94)	89 (85-92)	<.001
Minimum SBP, mm Hg	103 (93-114)	103 (93-114)	103 (92-114)	.36
Maximum heart rate, bpm	101 (90-115)	102 (90-116)	101 (90-114)	.14
Maximum respiratory rate, breaths/min	26 (20-34)	24 (20-32)	28 (22-38)	<.001
Maximum temperature, °C	37.5 (37.1-38.3)	37.4 (37.1-38.2)	37.7 (37.2-38.5)	<.001
Highest creatinine, mg/dL	0.85 (0.71-1.14)	0.85 (0.69-1.13)	0.87 (0.73-1.16)	<.001
Highest d-dimer, g/L	1 (0.6-1.9)	1.01 (0.6-2.08)	0.97 (0.58-1.76)	.008
Highest C-reactive protein, mg/L	72 (32-129)	66 (27.8-124)	80 (40-138)	<.001
Lowest absolute lymphocyte count, cells/μL	900 (600-1300)	900 (600-1300)	800 (600-1200)	.03
Inpatient data				
Mechanical ventilation	539 (9.7)	209 (6.1)	330 (15.9)	<.001
Admitted to ICU	1478 (26.9)	624 (18.2)	854 (41.1)	<.001
LOS, median (IQR), d				
Hospital	5.0 (2.8 − 9.2)	3.9 (2.2-6.7)	7.2 (4.5-13.8)	<.001
ICU	5.4 (2.0-12.5)	3.6 (1.4-9.1)	6.9 (2.8-15.3)	<.001

Abbreviations: ACE, angiotensin-converting enzyme; BMI, body mass index (calculated as weight in kilograms divided by height in meters squared); ICU, intensive care unit; LOS, length of stay; SBP, systolic blood pressure; Spo_2_, oxygen saturation as measured by pulse oximetry.

SI conversion factors: To convert C-reactive protein to milligrams per liter, multiply by 10; creatinine to micromoles per liter, multiply by 88.4; d-dimer to nanomoles per liter, multiply by 5.476; and lymphocytes to ×10^9^ per liter, multiply by 0.001.

^a^ Patients self-identified race, including other (overall category).

### Propensity-Matched Cohorts

**Figure 2.**
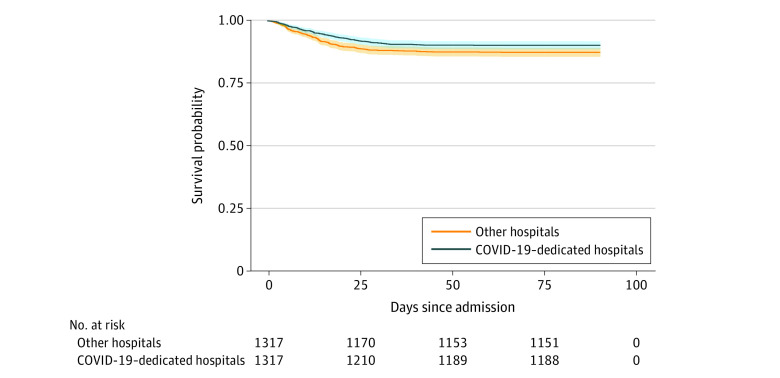
Survival for the Propensity Score–Matched Group of Patients Survival to discharge following propensity score matching was 90.2% in the COVID-19–dedicated hospital group compared with 88.4% in the other hospital group (*P* = .02).

Given the clear difference in severity in illness between the 2 groups, we performed propensity score matching to adjust for these differences. With propensity score matching, there were 1317 patients in both treatment groups. Propensity score graphs were comparable between the 2 groups (eFigure in the Supplement). The demographic, clinical, and laboratory data variables that were significantly different between the 2 groups were not significantly different after propensity score matching, apart from d-dimer levels (eTable 2 in the Supplement). Survival to discharge following propensity score matching was 90.2% in the COVID-19–dedicated hospital group compared with 88.4% in the other hospitals group. Kaplan-Meier survival curves for the propensity score–matched group are shown in Figure 2 with significantly improved in-hospital mortality rates in the COVID-19–dedicated hospital group (*P* = .02).

### Risk-Adjusted Outcomes

**Figure 3.**
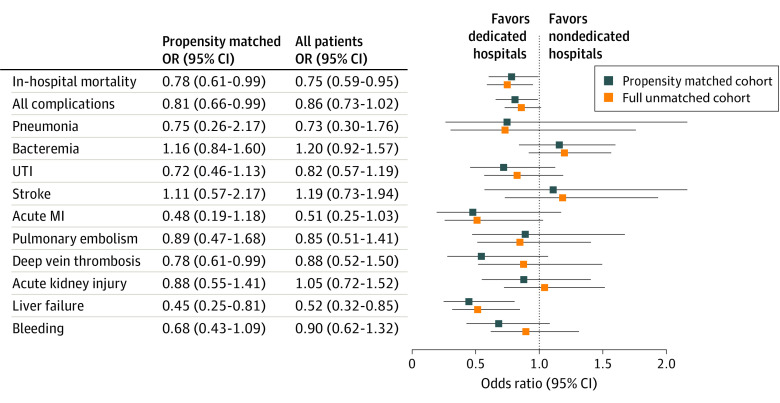
Adjusted Odds Ratios of In-Hospital Mortality and Common Complications in the Unmatched and the Propensity Score–Matched Cohort Adjusted odds ratios were calculated using logistic regression with risk adjustment. Full cohort was adjusted for age, non-English speaking, sex, body mass index, Elixhauser Comorbidity Index, home use of metformin, home use of insulin, oxygen saturation, systolic blood pressure, heart rate, respiratory rate, temperature, creatinine level, d-dimer level, C-reactive protein level, absolute lymphocyte count, tocilizumab administration, corticosteroid administration, deep vein thrombosis prophylaxis, admission month, mechanical ventilation, and intensive care unit admission. Adjustment for d-dimer level was performed in the propensity score–matched group. Calipers indicate 95% CIs. UTI indicates urinary tract infection.

Adjusted odds ratios (ORs) demonstrated a significantly decreased risk of in-hospital mortality for the patients treated at COVID-19–dedicated hospitals for both the unmatched group (OR, 0.75; 95% CI, 0.59-0.95) and the propensity score–matched group (OR, 0.78; 95% CI, 0.61-0.99) (Figure 3). The risk of all complications was significantly lower in the COVID-19–dedicated hospitals in the propensity score–matched group (OR, 0.81; 95% CI, 0.66-0.99). The rate of liver failure was the only individual complication that was significantly different at the COVID-19–dedicated hospitals and was decreased in both the full cohort (OR, 0.52; 95% CI, 0.32-0.85) and the propensity score–matched group (OR, 0.45; 95% CI, 0.25-0.81).

### Sensitivity Analyses

**Figure 4.**
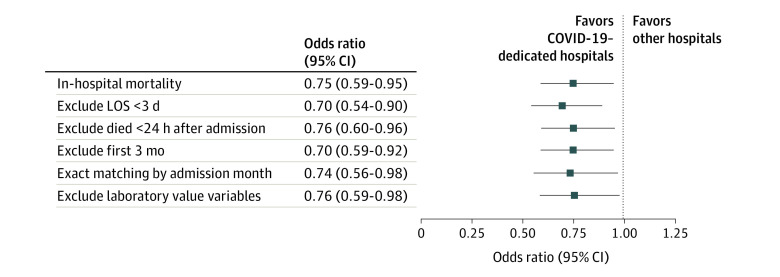
Sensitivity Analyses The first 3 analyses were performed on the full unmatched group with mortality risk adjusted for age, non-English speaking, sex, body mass index, Elixhauser Comorbidity Index, home use of metformin, home use of insulin, oxygen saturation, systolic blood pressure, heart rate, respiratory rate, temperature, creatinine level, d-dimer level, C-reactive protein level, absolute lymphocyte count, tocilizumab administration, corticosteroid administration, deep vein thrombosis prophylaxis, admission month, mechanical ventilation, and intensive care unit admission. The fourth analysis was performed by repeating propensity score matching with exact matching for admission month. In the final analysis, logistic regression was performed on the full unmatched cohort with the laboratory values excluded.

We opted to perform additional sensitivity analyses to further interrogate survival between the patient groups. All sensitivity analyses demonstrated improved survival in patients treated at a dedicated hospital (Figure 4). Three sensitivity analyses were performed on the full unmatched cohort and excluded patients with the following characteristics: hospital LOS 3 days or less (OR, 0.70; 95% CI, 0.54-0.90), patients treated within the first 3 months of the COVID-19–dedicated hospital’s operation (OR, 0.70; 95% CI, 0.53-0.92), and patients who died within 24 hours of admission (OR, 0.76; 95% CI, 0.59-0.96). A fourth analysis added exact matching by admission month to the propensity score matching to account for changes in treatment approaches over time (OR, 0.74; 95% CI, 0.56-0.98). A fifth sensitivity analysis performed the logistic regression on the full unmatched cohort while excluding the laboratory values because these accounted for most of the imputed data (OR, 0.76; 95% CI, 0.59-0.98).

### Quality Metrics and COVID-19 Therapeutics

The overall rate of DVT prophylaxis (either low molecular-weight heparin or unfractionated heparin) was 67.0%. Overall, COVID-19–specific therapeutics were used at a rate of 35.0% for high-dose corticosteroids, 50.9% for remdesivir, and 4.2% for tocilizumab. All the therapeutics as well as DVT prophylaxis were used at significantly higher rates in the COVID-19–dedicated hospitals. Deep vein thrombosis prophylaxis was administered in 83.9% of patients in COVID-19–dedicated hospital compared with 56.9% of those in other hospitals (*P* < .001), high-dose corticosteroids in 56.1% vs 22.2% (*P* < .001), remdesivir in 61.5% vs 44.5% (*P* < .001), and the anti-IL-6 antibody tocilizumab in 7.9% of vs 2.0% (*P* < .001) (eTable 3 in the Supplement).

## Discussion

The M Health Fairview health care system took a unique approach to handling the COVID-19 pandemic. We were able to rapidly create isolated COVID-19–dedicated hospitals designed to be focused on providing the most up-to-date treatments as well as ensuring the safety of the hospital staff.^7^ A major priority of the dedicated hospitals was constant evaluation of practice guidelines and close monitoring of patient outcomes. This report is a retrospective review of our experience in which we demonstrate that care in our dedicated hospitals was associated with improved mortality and increased adherence to emerging COVID-19–specific treatment options, supporting this model of care.

In our analysis, survival was significantly higher for patients treated at the COVID-19–dedicated hospitals using multiple methods of multivariate analysis, including logistic regression, propensity score matching, and sensitivity analyses. This patient population was more severely ill with older age and increased comorbidities, laboratory and physiologic abnormalities, and frequency of mechanical ventilation and ICU admission (Table). As a result, unadjusted survival was significantly decreased in the COVID-19–dedicated hospitals (Figure 1). After adjusting for the increased severity of illness, survival was significantly higher in both the unmatched (OR, 0.75; 95% CI, 0.59-0.95) and propensity score–matched (OR, 0.78; 95% CI, 0.58-0.99) groups. This difference was more pronounced in our sensitivity analyses when evaluating mortality in patients with hospital LOS longer than 3 days (OR, 0.70; 95% CI, 0.54-0.90). This finding is not surprising because patients who died from early complications of COVID-19 were likely to die despite any possible intervention, but those with prolonged hospitalization likely benefit from improved care provided by the COVID-19–dedicated hospitals. The overall in-hospital mortality in the patient population (9.4%) was in line with previously reported rates.^19,20^

The benefits of treatment at the COVID-19–dedicated hospitals were reflected in the rate of in-hospital complications as well. There was a significantly lower rate of overall complications in both the full cohort and the propensity score–matched groups with risk-adjusted logistic regression (Figure 3). Adherence to quality metrics, including use of high-dose corticosteroids, DVT prophylaxis, and therapeutics that were shown to be beneficial for treatment of COVID-19, further support the benefit of the dedicated hospitals. The use of all these interventions was significantly higher in the dedicated hospital group (*P* < .001), suggesting a more effective implementation of protocols and a more comprehensive and up-to-date understanding of the literature among physicians at the COVID-19–dedicated hospitals.

Numerous studies have reported that the volume of cases a center sees is associated with improved outcomes. High-volume centers have shown improved outcomes and survival for a range of individual procedures^21,22^ as well as broad disease categories, such as severe trauma^23^ and cancer.^21,22^ This improvement has been attributed, in part, to the volume of cases an individual proceduralist performs^24^ and the center’s patient volume, likely associated with the increased experience of the nursing and support staff.^21^ Kahn et al^25^ reported increased ICU volume and number of patients receiving mechanical ventilation treated annually was associated with improved outcomes. As a center with high volume dedicated solely to the care of patients with COVID-19, our dedicated hospitals benefited from a combination of these 2 factors. This improvement in understanding of the disease process is supported in our sensitivity analysis as the risk of in-hospital mortality at the dedicated hospitals was lower after removing all patients treated in the first 3 months of the pandemic (OR, 0.70; 95% CI, 0.53-0.92). Given that we adjusted for COVID-19–specific treatments in our model, this improvement in mortality likely comes in part from intangible and unmeasurable variables. These factors potentially include early identification of multiorgan failure and appropriate treatment, timely transfer to higher levels of care, and improved understanding by the hospital staff of the disease process. The separation of mortality rates shown in Figure 2 occurred in the first 10 to 15 days, suggesting the benefit of the dedicated hospitals is most prominent in the treatment given early in the hospitalization. Other previously undescribed factors may also play a role, such as decreased health care professional cognitive load when using personal protective equipment for all patients, decreased anxiety from universal personal protective equipment use, increased health care professional well-being from the COVID-19–dedicated hospitals’ unique camaraderie, and other factors. In addition, the creation of COVID-19–specific hospitals provided the benefit of isolating patients with infection to decrease potential in-hospital spread, as proven by the low rate of transmission from patient-to-clinicians at both sites.^13^

### Limitations

There were limitations of this study. Although the data were collected prospectively, the analysis was performed retrospectively. There were also missing data for several of the laboratory variables, affecting the ability to perform risk-adjusted analyses and propensity score matching. For this reason, imputation had to be performed to create a complete data set. These limitations have been outlined extensively.^26,27^ In addition, the outcome of many nursing cares that have been associated with improved mortality, such as prone positioning, could not be extracted from the database, which may have had an unquantified effect on mortality. Staffing differences were difficult to account for as well, which also may have affected mortality. There also was clearly bias in the patients who were selected for transfer to the COVID-19–dedicated hospitals by the clinicians at the admitting hospital. These patients were significantly more ill (Table). The code status of the patients was not always fully documented and could not be included in the analysis. Therefore, information regarding advanced directives was not included in the dedicated hospital group and may have been associated with the decreased survival in the other hospital group. It is unclear whether this association with improved mortality would be found in dedicated COVID-19 wards within hospitals because many of the factors outlined would also be present in this setting. This issue represents an additional interesting area of future study.

## Conclusions

In this cohort study, we identified a mortality benefit associated with being cared for at a COVID-19–dedicated hospital vs other hospitals. The dedicated hospitals also administered more COVID-19–specific treatments with rapid implementation of new processes of care and were associated with significantly fewer overall complications. Given the ongoing globalization and increasing rate of zoonosis, the risk of viral pandemics will likely only continue to increase.^28,29^ In the event of future outbreaks of viral pneumonia or other infectious diseases, we believe that the success of our model could provide a potential framework for large health care organizations looking to isolate patients with infections and provide the best care for these individuals.

## Notes

Supplement. **eTable 1.** Variables Associated With In-Hospital Mortality **eTable 2.** Demographic, Clinical, and Laboratory Data **eTable 3.** COVID-19 Therapeutic Measure **eFigure.** Propensity Scores of Matched Cohort
