# Supplementary material for: Comparison of Outcomes and Process of Care for Patients Treated at Hospitals Dedicated for COVID-19 Care vs Other Hospitals
Source: JAMA Netw Open. 2022 Mar 3;5(3):e220873. doi: 10.1001/jamanetworkopen.2022.0873 (PMC8895262; doi:10.1001/jamanetworkopen.2022.0873)
Supplement: Supplement. — eTable 1. Variables Associated With In-Hospital Mortality eTable 2. Demographic, Clinical, and Laboratory Data eTable 3. COVID-19 Therapeutic Measure eFigure. Propensity Scores of Matched Cohort [file jamanetwopen-e220873-s001.pdf]

Supplementary Online Content

Bergman ZR, Usher M, Olson A, et al. Comparison of outcomes and process of care for patients treated at hospitals dedicated for COVID-19 care vs other hospitals. *JAMA Netw Open*. 2022;5(3):e220873. doi:10.1001/jamanetworkopen.2022.0873

eTable 1. Variables Associated With In-Hospital Mortality

eTable 2. Demographic, Clinical, and Laboratory Data

eTable 3. COVID-19 Therapeutic Measure

eFigure. Propensity Scores of Matched Cohort

This supplementary material has been provided by the authors to give readers additional information about their work.

© 2022 Bergman ZR et al. *JAMA Network Open.*

eTable 1. Variables Associated With In-Hospital Mortality

Proposed Variables by Intensive Care Providers	LASSO Variables Associated with Mortality
Age	Age
Race/Ethnicity	Language
Language	Gender
Gender	Body Mass Index
Body Mass Index	Elixhauser Comorbidity Index
Elixhauser Comorbidity Index	Home Metformin
Home Loop Diuretic	Home Insulin
Home ACE inhibitor	Minimum Oxygen Saturation (first 48 hours)
Home Metformin	Minimum Systolic Blood Pressure (first 48 hours)
Home Insulin	Maximum Heart Rate (first 48 hours)
Home Inhaled Steroid	Maximum Respiratory Rate (first 48 hours)
Minimum Oxygen Saturation (first 48 hours)	Maximum Temperature (first 48 hours)
Minimum Systolic Blood Pressure (first 48 hours)	Highest Creatinine (first 72 hours)
Maximum Heart Rate (first 48 hours)	Highest C-Reactive Protein (first 72 hours)
Maximum Respiratory Rate (first 48 hours)	Highest Absolute Lymphocyte Count (first 72 hours)
Maximum Temperature (first 48 hours)	Tocilizumab Administration
Highest Creatinine (first 72 hours)	High Dose Steroids Administration
Highest D-dimer (first 72 hours)	Received Appropriate DVT Prophylaxis
Highest C-Reactive Protein (first 72 hours)	Mechanical Ventilation
Highest Absolute Lymphocyte Count (first 72 hours)	ICU Admission
Remdesivir Administration	Month of Admission
Tocilizumab Administration	
High Dose Steroids Administration	
Received Appropriate DVT Prophylaxis	
Mechanical Ventilation	
ICU Admission	
Month of Admission	

eTable 1. The left column contains clinical and demographic variables selected by the consensus of seven Intensive Care Providers at the COVID-19 Cohort hospitals that were likely to be associated with mortality outcomes. The right column contains the variables that were identified to be associated with mortality in our model developed with the use of the Least absolute shrinkage and selection operator (LASSO) method.

© 2022 Bergman ZR et al. *JAMA Network Open.*

eTable 2. Demographic, Clinical, and Laboratory Data

Variables		Non-Cohort N=1,317	Cohort N=1,317	*p*-value
Age		64.6(48.4-76.5)	63.0 (49.6-76.3)	0.68
BMI		890 (67.6)	839 (63.7)	0.20
Non-English Speaking		225 (17.1)	226 (17.2)	0.39
Race (n, [%])	Asian	81 (6.2)	98 (7.4)	0.67
	Black	151 (11.5)	172 (13.1)	
	Hispanic	27 (2.1)	19 (1.4)	
	White	152 (11.5)	168 (12.8)	
	Declined	16 (1.2)	21 (1.6)	
	Other	29.57 (25.46-34.1)	29.4 (24.7-34.7)	
Sex (n, [%])	Female	645 (49.0)	652 (49.5)	0.78
	Male	672 (51.0)	665 (50.5)	
Comorbidities/Home Medications (n, [%])	Elixhauser Comorbidity Index (median [IQR])	6 (3-9)	6 (3-9)	0.63
	Home ACE Inhibitor	159 (12.1)	129 (9.8)	0.061
	Home Metformin	94 (7.1)	100 (7.6)	0.65
	Home Insulin	122 (9.3)	116 (8.8)	0.68
	Home Inhaled Steroid	107 (8.1)	130 (9.9)	0.12
Admission Data within 48 hours (median [IQR])	Minimum Oxygen Saturation (SpO_2)	89 (85-93)	89 (85-92)	0.99
	Minimum SBP (mmHg)	102 (91-113)	104 (93-114)	0.07
	Maximum Heart Rate (BPM)	102 (90-116)	101 (90-115)	0.36
	Maximum Respiratory Rate (breaths/min)	28 (20-36)	27 (22-35)	0.77
	Maximum Temperature (Fahrenheit)	99.6 (98.8-101)	99.6 (98.8-100.9)	0.45
	Creatinine (mg/dL)	0.85 (0.7-1.15)	0.86 (0.72-1.17)	0.12
	D-dimer (g/L)	1 (0.6-2.1)	0.92 (0.54-1.76)	**0.003**
	C-Reactive Protein (mg/L)	72.3 (34-131)	72 (31-127)	0.46
	Absolute Lymphocyte Count (Kcells/uL)	0.9 (0.6-1.2)	0.9 (0.6-1.3)	0.87
Inpatient Data (n, [%])	Tocilizumab	57 (4.3)	48 (3.6)	0.37
	High Dose Steroids	650 (49.4)	610 (46.3)	0.12
	DVT prophylaxis	969 (73.6)	1,002 (76.1)	0.14
	Mechanical Ventilation	155 (11.8)	133 (10.1)	0.17
	Admitted to ICU	409 (31.1)	368 (27.9)	0.080

eTable 3. Patient demographics, admission data, and inpatient data in the propensity matched cohort. Variables that were significant different between the two groups were no longer significant after matching with the exception of D-dimer levels. Patients self-identified race, including “Other”. ACE – Angiotensin Converting Enzyme, BPM – Beats Per Minute, DVT – Deep Vein Thrombosis, ICU – Intensive Care Unit

© 2022 Bergman ZR et al. *JAMA Network Open.*

eTable 3. COVID-19 Therapeutic Measures

Medications		All admitted patients N=5,504		Non-Cohort N=3,427		Cohort N=2,077		*p*-value
		n	%	n	%	n	%	
Steroid Administration	Low	77	1.3	56	1.5	21	1.0	0.086
	High	1,928	35.0	762	22.2	1,166	56.1	**<0.001**
Remdesivir		2,803	50.9	1,526	44.5	1,277	61.5	**<0.001**
Tocilizumab		233	4.2	68	2.0	165	7.9	**<0.001**
DVT Prophylaxis – Either unfractionated or LMW heparin		3,691	67.0	1,949	56.9	1,742	83.9	**<0.001**

eTable 3. Rate of COVID-19 therapeutic use in full patient cohort with significantly higher use of literature supported therapeutic use in the COVID-19 Cohort hospitals. DVT – Deep Vein Thrombosis, LMW- Low Molecular Weight

© 2022 Bergman ZR et al. *JAMA Network Open.*

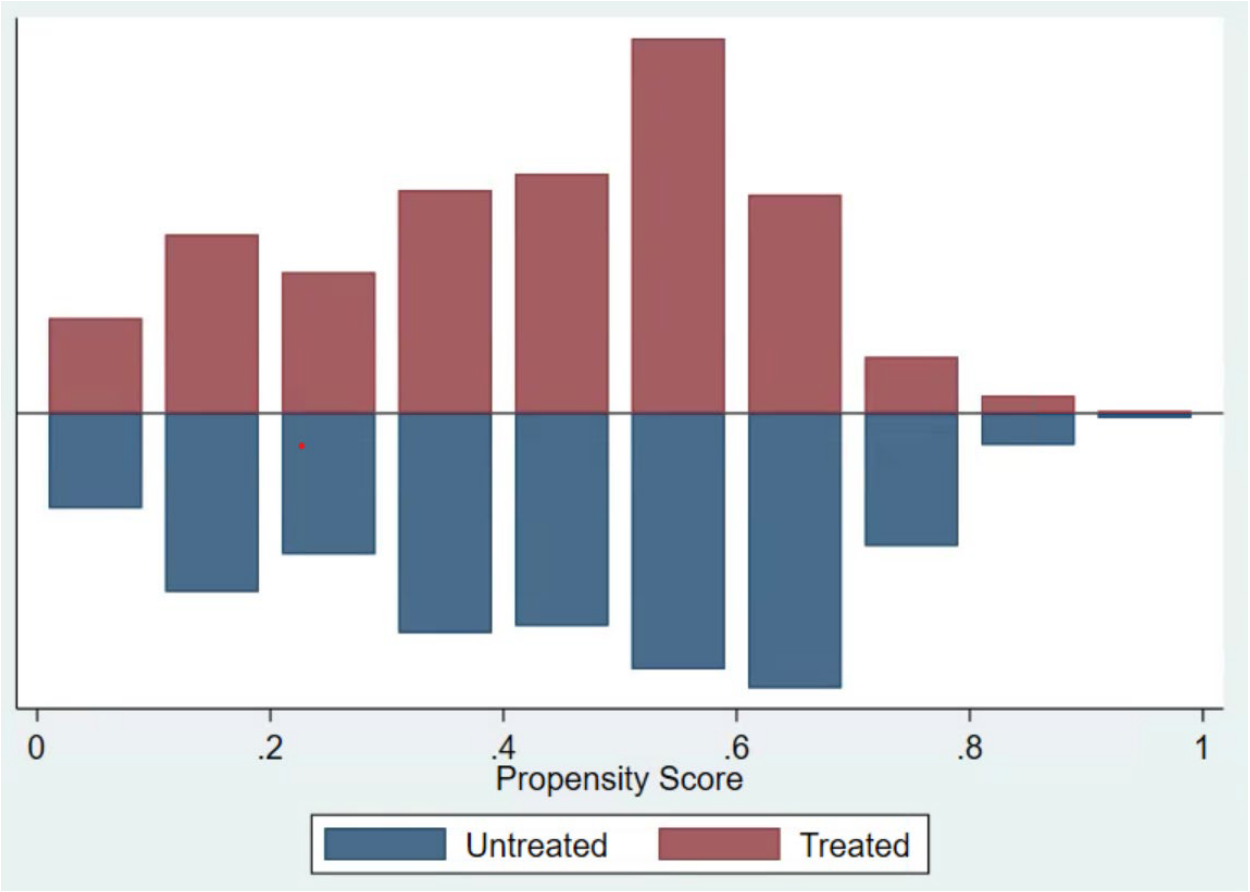

eFigure. Propensity scores of matched cohort. The treated group are patients that received care at the COVID-19 hospitals compared to those that did not in the untreated group.

© 2022 Bergman ZR et al. *JAMA Network Open.*
